# Cardioprotective Effect of Saffron Extract and Safranal in Isoproterenol-Induced Myocardial Infarction in Wistar Rats

**Published:** 2013-01

**Authors:** Roya Mehdizadeh, Mohammad–Reza Parizadeh, Ali-Reza Khooei, Soghra Mehri, Hossein Hosseinzadeh

**Affiliations:** 1 Department of Pharmacodynamics and Toxicology, School of Pharmacy, Mashhad University of Medical Sciences, Mashhad, Iran; 2 Department of Biochemistry and Nutrition, Faculty of Medicine, Mashhad, Iran; 3 Department of Pathology, Imam Reza Hospital, Faculty of Medicine, Mashhad, Iran; 4 Pharmaceutical Research Center, Department of Pharmacodynamics and Toxicology, School of Pharmacy, Mashhad University of Medical Sciences, Mashhad, Iran

**Keywords:** Crocus sativus, Isoproterenol, Lipid peroxidation, Myocardial infarction, Oxidative stress, Saffron, Safranal

## Abstract

***Objective(s):*** This study was designed to evaluate the cardioprotective effect of *Crocus sativus *L. (saffron) aqueous extract and safranal, the major constituent of the essential oil of saffron, on lipid peroxidation, biochemical parameters and histopathological findings in isoproterenol (ISO)-induced myocardial infarction in Wistar rats.

***Materials and Methods:*** The saffron extract (20, 40, 80 and 160 mg/kg/day IP) or control were administered for 9 days along with ISO (85 mg/kg, SC, at 24 hr interval) on 8th and 9th day in rats. Activities of creatine kinase-muscle, brain (CK-MB) and lactate dehydrogenase (LDH) were measured using standard commercial kits. The level of malondialdehyde in heart tissue was estimated with thiobarbituric acid reactive species test. For histopathological examination, hematoxylin and eosin (H&E) staining was used.

***Results:*** ISO administration induced a statistically significant increase (*P<* 0.001) in serum LDH and CK-MB and a significant increase (*P<* 0.001) in the levels of thiobarbituric acid reactive substances (TBARs) in the heart as compared to vehicle control rats. Saffron pretreatment (20, 40, 80 and 160 mg/kg IP) or safranal pretreatment (0.025, 0.050, 0.075 ml/kg IP) for 8 days, significantly decreased (*P<* 0.001) the serum LDH and CK-MB and myocardial lipid peroxidation as compared to ISO- induced rats. Histological findings of the heart sections confirmed myocardial injury with ISO administration and preserved nearly normal tissue architecture with saffron or safranal pretreatment.

***Conclusion:*** Saffron and safranal may have cardioprotective effect in ISO-induced myocardial infarction through modulation of oxidative stress in such a way that they maintain the redox status of the cell.

## Introduction

Myocardial infarction (MI), an acute condition of myocardial necrosis due to imbalance between coronary blood supply and myocardial demand, is regularly followed by several biochemical alterations such as lipid peroxidation, hyperlipidemia, free radical damage and hyperglycemia leading to qualitative and quantitative alterations of myocardium (1).

Treatment of ischemic injury includes restoration of blood supply to ischemic tissue and preventing the damage inflicted at the time of injury. Increasing reactive oxygen species like hydroxyl radicals (OH) and superoxide anion (O_2_^−^ ) during heart ischemia leads to destruction of cell membrane, development of lipid peroxides and damage of antioxidative defense system (2, 3). Experimental and clinical studies have shown that infarct size of myocardial necrosis can be limited by development of endogenous antioxidant enzymes and suppression of free radical generation (4). 

Isoproterenol, a synthetic non-selective β adrenoceptor agonist, has been recognized to induce myocardial infarction in rats as a result of disturbed physiological balance between formation of free radicals and antioxidative defense system (5, 6). During myocardial necrosis in acute condition, lipid peroxidation is increased and activities of cardiac injury marker and antioxidant enzymes are altered (3, 7). Myocardial infarction of isoproterenol-treated rats has been found to be similar to human MI in pathophysiological and morphological changes (8).

*Crocus sativus* L. commonly known as saffron is a stemless herb of the Iridaceae family. Its pharmacologically active and important constituents are safranal, crocin, picrocrocin and crocetin (9). 

Safranal is a monoterpene aldehyde which is the major constituent of the essential oil of saffron and is responsible for the saffron odor and aroma (10, 11). Safranal is formed in saffron by hydrolysis from picrocrocin (10).

Saffron and its constituents are widely evaluated for their pharmacological activities such as, antidepressant (12), anticonvulsant (13), antitussive (14), aphrodisiac activity (15), antianxiety, hypnotic (16), bronchodilator (17-18) and especially for their antitumor effect (10). 

Saffron and its constituents reduced lipid peroxidation in renal (19), hippocampal (20) and muscle skeletal (21) homogenates during ischemia-reperfusion-induced oxidative damage in rats. Radical scavenging activity of *C. sativus *L. extract and its bioactive constituents, safranal and crocin have been shown using DPPH (1,1-diphenyl-2-picryl-hydrazyl) radical scavenging test (22), deoxyribose assay and microsomal lipid peroxidation induced by Fe^2+^/ascorbat (23).

Therefore the aim of this study was to evaluate the cardioprotective effect of saffron and safranal on lipid peroxidation, biochemical and histopathological changes in ISO-induced oxidative myocardial damage in Wistar rats. 

## Materials and Methods

***Animals***

Male Wistar albino rats weighing 200–250 g were obtained from the Central Animal House Facility of BUALI Institute of Medical Sciences, Mashhad. Animals were held in the departmental animal house under controlled situation of temperature of 25±2°C, qualified humidity of 60 ± 5% and light–dark cycle of 12:12 hr. Animals were kept in polypropylene cages, each including a maximum of six animals.

***Drugs and chemicals***

Isoproterenol hemisulphate was dissolved in 0.9% saline and was applied within 10 min of preparation. Creatine kinase-MB (CK-MB) isoenzyme detection kit was obtained from Logotech India Pvt. Ltd. (Delhi, India). All chemicals used in this research were of analytical score and bought from Sigma Chemicals (St. Louis, MO, USA).

***Preparation of saffron extract***

Saffron (*C. sativus* L.) stigmas were collected from Ghain (Khorasan province, Iran). Dried stigmas were sliced and macerated in water for 3 days. Then the combination was filtered and prepared solution incorporated 100 mg/ml concentration from dry weight of stigma. This method was selected based on the recent used pattern among consumers.

***Induction of experimental myocardial infarction***

Isoproterenol (85 mg/kg) was administered SC to rats daily for two successive days on days 8th and 9th respectively to induce experimental myocardial infarction (24). 

***Experimental groups***

A total of 72 animals were used in this study. They were randomly divided into 12 groups, with 6 rats in each group.

*Group 1 (vehicle control)*

Rats received only normal saline for 9 days.

*Group 2 (ISO-control)*

Rats received normal saline for 9 days and were administered with 0.3 ml of isoproternol SC on the 8th and 9th days at an interval of 24 hr.

*Groups 3 to 6 (* *Aqueous extract* *of saffron* *+isoproterenol)*

Animals were pretreated with aqueous extract (20, 40, 80 and 160 mg/kg/day) IP for 9 days and on the 8th and 9th days received isoproternol SC at an interval of 24 hr.

*Groups 7 to 9 (* *Safranal* *+isoproterenol)*

Animals were pretreated with safranal (0.025, 0.050 and 0.075 ml/kg/day) IP for 9 days and on the 8th and 9th days isoproterenol was administered SC at an interval of 24 hr.

*Group 10 (Aqueous extract per se)*

Animals were treated with aqueous extract (160 mg/kg/day) IP for 9 days and on the 8th and 9th days 0.3 ml saline was administered SC at an interval of 24 hr.

*Group 11 (Safranal per se)*

Animals were treated with safranal (0.075 ml/kg/day) IP for 9 days and on the 8th and 9th days 0.3 ml saline was administered SC at an interval of 24 hr.

*Group 12 (* *vit E* *+isoproterenol)*

Animals were pretreated with vit E IP for 9 days and on the 8th and 9th days 0.3 ml saline was administered SC at an interval of 24 hr.

Changes in bodyweight, food and water intake outline of animals in all groups were noted during the experimental at standard intervals.

***Marker enzyme assays***

The marker enzymes LDH and CPK were assayed in serum using standard kits. The results were expressed as IU/l for LDH and CPK.

***Lipid peroxidation***

Animals of all groups were sacrified by decaritation. Their hearts were excised and processed for biochemical and histopathological studies. For biochemical analysis, hearts were removed and stored in liquid nitrogen, whereas for light microscopic studies they were fixed in 10% buffered formalin. 

A marker of lipid peroxidation, Malondialdehyde (MDA) levels were measured in heart tissue. MDA reacts with thiobarbituric acid (TBA) as a thiobarbituric acid reactive substance (TBARS) to produce a red colored complex which has peak absorbance at 532 nm (21). 

Three ml phosphoric acid (1%) and 1 ml TBA (0.6%) was added to 0.5 ml of heart homogenate 10% in a centrifuge tube and the mixture was heated for 45 min in a boiling water bath. After cooling, 4 ml of n-butanol was added to the mixture and was vortex-mixed for 1 min followed by centrifugation at 70000 rpm for 20 min. The organic layer was transferred to a fresh tube and its absorbance was measured at 532 nm. MDA levels were expressed as nmol/g tissue.

***Histopathological study (Light Microscope)***

Animals were sacrificed on the defined days, and immediately their hearts were completely and intactly expelled, washed with saline and instantly fixed in 10% buffered formalin. The hearts were stored in formalin overnight and then every globe was horizontally sectioned at about 2-3 mm thickness by the Automatic Tissue Processor (Sacuta, Tokyo, Japan). All the sections were embedded in paraffin blocks separately, sections cut at 5 µm by Leica 2BS Microtome (Deutsch, Landy) and stained with hematoxylin and eosin (H&E). These sections were then examined under Light Microscope (Olympus BX 40, Tokyo, Japan). The pathologist performing histopathologic study was blinded to the treatment assignment of various study groups. The pathological scores were done as following: (0)=no damage; (1)= damage to the 25% thickness of the myocardium; damage to the 50% thickness of the myocardium; damage to the 75% thickness of the myocardium (4)= damage to the full thickness of the myocardium. 

**Figure 1:**
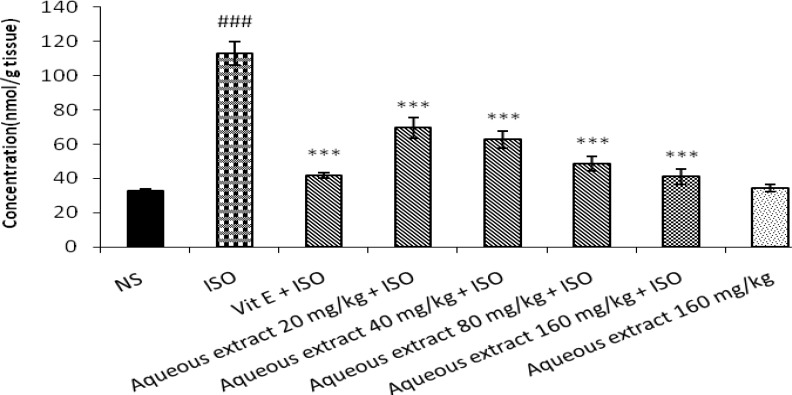
Effect of aqueous extract of saffron on level of malondialdehyde (MDA) in isoproterenol induced myocardial infarction in rats. NS: Normal saline, ISO: isoproterenol. All values are expressed as mean±S.D. for each group (n=8/group). Significance was determined by One-Way ANOVA followed by Tukey-Kramer test. ###*P<*0.001 v.s vehicle treated ***P<0.001 versus isoproterenol-control

***Statistical analyses***

All data was expressed as mean±SD. Significance was determined by One-Way ANOVA followed by Tukey-Kramer test. 

Kruskal-Wallis test was used to compare histopathological data (non-parametric data). *P<* 0.05 was recognized as statistically significant. 

## Results

***Effect of*** ***aqueous extract of saffron*** ***and safranal on cardiac injury markers***

As shown in Table 1 and 2, treatment with isoproterenol significantly increased activities of CK-MB and LDH in the serum in comparison with control (*P<* 0.001). Pretreatment with aqueous extract of saffron (80 and 160 mg/kg) or safranal (0.050 and 0.075 ml/kg) daily for a period of 9 days normalized the activities of these enzymes in serum compared to ISO alone treated rats (*P<*0.001). 

**Table 1:** Effect of aqueous extract of saffron on CK-MB and LDH activities in isoproterenol induced myocardial infaraction in CK-MB

Treatment groups	CK-MB ( IU/L )	LDH ( IU/L )
vehicle control	9205.83± 1252.56	2050.83±98.39
ISO-control	22653.67±1681.23^###^	3439.83±116.42^###^
Aqueous extract 20 mg/kg + ISO	20561.83±1141.36	3143.88±88.66
Aqueous extract 40 mg/kg + ISO	16695.16±2327.25	2986.66±69.81
Aqueous extract 80 mg/kg + ISO	13276.73±1641^***^	2626.16±126.29^***^
Aqueous extract 160 mg/kg + ISO	11779.1667±815.38^***^	2496.33±124.18^***^
Vit E + ISO	12839.83±423.54^***^	2594.5±111.24852^***^
Aqueous extract 160 mg/kg	10755.167±1212.96	2196.667±109.06

CK-MB: creatine kinase-MB, LDH: lactate dehydrogenase, NS: Normal saline, ISO: isoproterenol. All values are expressed as mean±S.D. for each group (n=6/group). Significance was determined by One-Way ANOVA followed by Tukey-Kramer test: ### *P<* 0.001 v.s vehicle treated; *** *P<*0.001 v.s isoproterenol-control

**Table 2:** Effect of safranal on CK-MB and LDH activities in isoproterenol induced myocardial infaraction in CK-MB

Treatment groups	CK-MB ( IU/l )	LDH ( IU/l )
vehicle control	9205.83± 1252.56	2050.83±98.39
ISO-control	22653.67±1681.23^###^	3439.83±116.42^###^
Safranal 0.025 ml/kg + ISO	16012.16±1605.87^*^	2805.33±115.90^**^
Safranal 0.050 ml/kg + ISO	13134.16±1017.33^***^	2633.78±102.97^***^
Safranal 0.075 ml/kg + ISO	10254.66±1408.67^***^	2429.66±92.82^***^
Vit E + ISO	12839.83±423.54^***^	2594.5±111.24852^***^
Safranal 0.075 ml/kg	11100.5±1248.03	2097.66±111.53

CK-MB: creatine kinase-MB, LDH: lactate dehydrogenase, NS: Normal saline, ISO: isoproterenol. All values are expressed as mean±S.D. for each group (n=8/group). Significance was determined by One-Way ANOVA followed by Tukey-Kramer test: ### *P<*0.001 v.s vehicle treated; *** *P<*0.001, ** *P<* 0.01 and *P<0.05 versus isoproterenol-control

***Effect of aqueous extract of saffron and*** ***safranal*** ***on lipid peroxidation***

**Figure 2:**
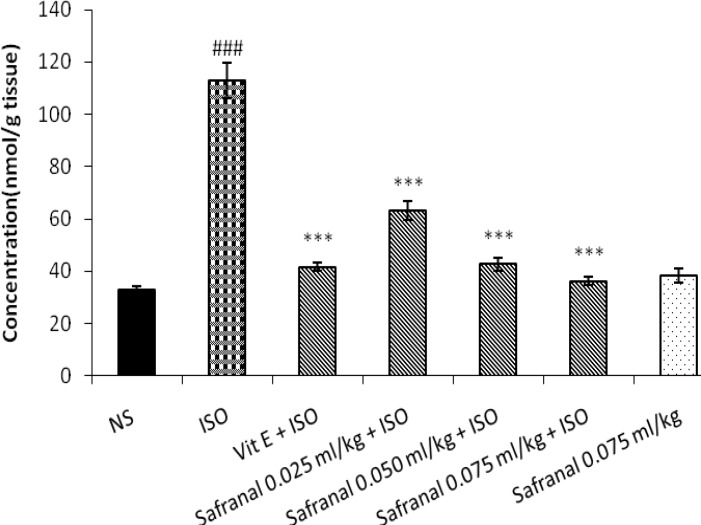
Effect of Safranal on level of malondialdehyde (MDA) in isoproterenol induced myocardial infarction in rats. NS: Normal saline, ISO: isoproterenol. All values are expressed as mean±SD for each group (n= 8/group). Significance was determined by One-Way ANOVA followed by Tukey-Kramer test: ##*P<*0.001 v.s vehicle treated, ** ***P<0.001 v.s isoproterenol-control

Isoproternol significantly increased level of MDA as a marker of lipid peroxidation in heart tissue in treated rats in comparison with control (*P< *0.001). As shown in Figure 1 and 2, pretreatment with aqueous extract of saffron (20, 40, 80 and 160 mg/kg) or safranal (0.025, 0.050, 0.075 ml/kg) could reduce lipid peroxidation and level of MDA in heart tissue when compared with isoproternol alone treated rats (*P<*0.001). The aqueous extract (160 mg/kg) or safranal (0.075 ml/kg) alone did not show any significant changes in lipid peroxidation. 

**Figure 3A:**
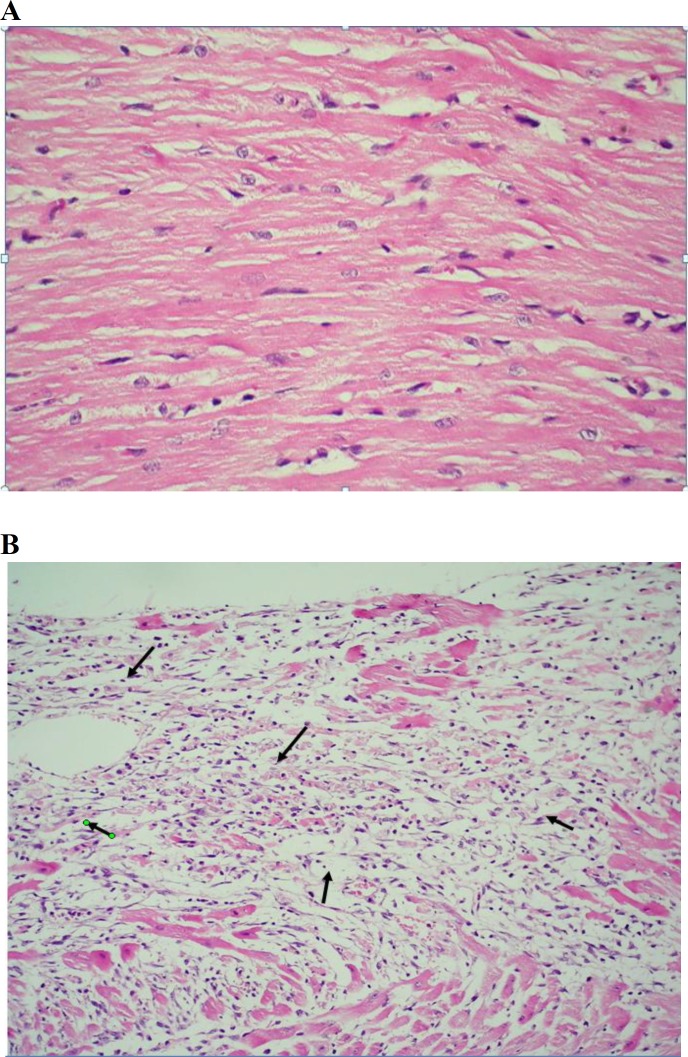
Light micrograph of vehicle treated rat heart showing normal architecture of myocytes (H&E, 200×) Figure 3B. Light micrograph of isoproterenol-control group heart showing (→) focal confluent necrosis of muscle fibers with inflammatory cell infiltration and edema (H&E, 100×)

***Histopathological examination of cardiac tissues***

In histopathological examination, tissue necrosis, interstitial edema, leukocyte infiltration and vascular changes were noted. 

**Table 3:** Effect of aqueous extract of saffron and safranal on % myocardium injury in isoproterenol induced myocardial infaraction in rats

Treatment groups	% Myocardium injury
NS	0
ISO-control	75 ±12
Aqueous extract 20 mg/kg + ISO	58 ± 6
Aqueous extract 40 mg/kg + ISO	50 ± 12
Aqueous extract 80 mg/kg + ISO	16 ± 13
Aqueous extract 160 mg/kg + ISO	8 ± 6
Safranal 0.025 ml/kg + ISO	66 ± 6.9
Safranal 0.050 ml/kg + ISO	41 ± 6
Safranal 0.075 ml/kg + ISO	25 ± 12
Vit E + ISO	41 ± 6

NS: Normal salin, ISO: isoproterenol. All values are expressed as mean±SEM for each group (n=3/group). Statistical analyses of experimental values were performed with Kruskal-Wallis

The severity of these alterations was scored in a semiquantitive manner for each group. Tissue sections from hearts of rats treated with ISO showed extensive myocardial coagulative necrosis, edema and leukocyte infiltration especially in ventricular walls (Figure 3B) as compared to control group. The sections of hearts from pretreated rats with aqueous extract of saffron (20, 40, 80 and 160 mg/kg) (Figure 4) or safranal (0.025, 0.050, 0.075 ml/kg) exhibited much less such alteration which were merely observed scattered.

In control group, heart tissues showed no considerable histopathologic changes (Figure 3A). 

## Discussion

Isoproterenol is extensively used as a model of assessing cardioprotective drugs and studying myocardial consequences of ischemic disorders due to supramaximal doses of isoproterenol stimulate subendocardial myocardial ischemia, necrosis, hypoxia, and at last fibroblastic hyperplasia with decreased myocardial compliance and inhibition of diastolic and systolic function, which is strongly similar to local myocardial infarction-like pathological changes seen in human myocardial infarction (8). 

In this study, we found that saffron and safranal protected myocardium from isoproterenol-induced myocardial functional and structural damage through reduction of lipid peroxidation.

When the cell membrane becomes permeable or rupture, lactate dehydrogenase, and creatine kinase spread out from the damaged tissues to the blood stream and serve the diagnostic markers of myocardial tissue injury (25). The quantity of these cellular enzymes existing in plasma reflects the changes in plasma membrane integrity and/or permeability. 

Drugs like silibinin, squalene and naringin stabilize cell membrane by a decline in lactate dehydrogenase and creatine kinase levels (25-28). 

According to the results of present study, isoproterenol significantly increased levels of lactate dehydrogenase and creatine kinase and necrotic damage in the myocardial membrane.

The prior administration of saffron was found to considerably reduce the isoproterenol-induced raise in the activities of lactate dehydrogenase, and creatine kinase. 

Protective effect of saffron and its main constituent crocin and safranal have been shown in different *in vivo* and *in vitro* models (23, 28). It was shown that saffron and its constituents could decrease lipid peroxidation in renal (19), hippocampal (20) and muscle skeletal (21) homogenates during ischemia-reperfusion-induced oxidative damage in rats. 

Administration of isoproterenol had been reported to induce oxidative stress and necrotic injuries in the myocardium of rats (5).

The generation of reactive oxygen species and/or reduction of the antioxidants in the protective system may contribute to oxidative stress and influence the pathogenesis of myocardial infarction (29). 

The results presented in this study showed that pretreatment with safranal (0.025, 0.05, 0.075 ml/kg) or the aqueous extract (20, 40, 80 and 160 mg/kg) could reduce level of malondialdehyde content as a marker of lipid peroxidation. 

High antioxidant capacity of saffron has been studied (22, 23). With regards to other studies it seems that at least one part of cadioproecive effect of saffron extract and safranal in isoprterenol–induced cardiotoxicity is due to antioxidant activity. 

Histopathologic examination revealed that pretreatment with safranal (0.025, 0.05 and 0.075 ml/kg) and the aqueous extract (20, 40, 80 and 160 mg/kg) as compared to control group yielded strong protection of myocardial cells from cellular tissue injury produced with ISO- treatment.

## Conclusions

In conclusion, the present study showed that pretreatment with saffron or safranal reduced histopathological changes in heart tissue and decreased CK-MB and LDH activities in serum. Saffron and safranal could also reduce lipid peroxidation in heart tissue. It seems that saffron’s cardioprotective effect in this model was partly modulated via reduction of oxidative stress. 
